# Heterogeneous Ensemble-Based Spike-Driven Few-Shot Online Learning

**DOI:** 10.3389/fnins.2022.850932

**Published:** 2022-05-09

**Authors:** Shuangming Yang, Bernabe Linares-Barranco, Badong Chen

**Affiliations:** ^1^School of Electrical and Information Engineering, Tianjin University, Tianjin, China; ^2^Microelectronics Institute of Seville, Seville, Spain; ^3^Institute of Artificial Intelligence and Robotics, Xi’an Jiaotong University, Xi’an, China

**Keywords:** spiking neural network, few-shot learning, entropy-based learning, spike-driven learning, brain-inspired intelligence

## Abstract

Spiking neural networks (SNNs) are regarded as a promising candidate to deal with the major challenges of current machine learning techniques, including the high energy consumption induced by deep neural networks. However, there is still a great gap between SNNs and the few-shot learning performance of artificial neural networks. Importantly, existing spike-based few-shot learning models do not target robust learning based on spatiotemporal dynamics and superior machine learning theory. In this paper, we propose a novel spike-based framework with the entropy theory, namely, heterogeneous ensemble-based spike-driven few-shot online learning (HESFOL). The proposed HESFOL model uses the entropy theory to establish the gradient-based few-shot learning scheme in a recurrent SNN architecture. We examine the performance of the HESFOL model based on the few-shot classification tasks using spiking patterns and the Omniglot data set, as well as the few-shot motor control task using an end-effector. Experimental results show that the proposed HESFOL scheme can effectively improve the accuracy and robustness of spike-driven few-shot learning performance. More importantly, the proposed HESFOL model emphasizes the application of modern entropy-based machine learning methods in state-of-the-art spike-driven learning algorithms. Therefore, our study provides new perspectives for further integration of advanced entropy theory in machine learning to improve the learning performance of SNNs, which could be of great merit to applied developments with spike-based neuromorphic systems.

## Introduction

The human brain has the advantages of imagination, lifelong learning, and learning based on the interaction with the environment. Especially, the human brain can learn a new concept from a small number of examples and has the strong generalization capability, which outperforms current machine intelligence (Goelet et al., 1986). Some extraordinary capabilities exist in the human brain. For example, when giving a reference example, the brain can be easily generalized to new examples or create a new example. It is necessary and meaningful to develop a novel brain-inspired framework to break the current bottleneck of machine intelligence based on brain processing and learning mechanism.

A spiking neural network (SNN) is the third generation of an artificial neural network (ANN), which is based on the underlying mechanism of the biological brain (Falez et al., 2019; Paredes-Vallés et al., 2019). It has the advantages of rich spatiotemporal dynamical characteristics, large diversities of the neural encoding mechanism, and low-power event-based computation (Yang et al., 2021a,b). It is critical and meaningful for artificial general intelligence (AGI), and is essential for high-efficiency edge computing devices with low power consumption and real-time processing capability (Pei et al., 2019).

In recent years, along with the development of computing devices, deep learning with a large amount of labeled data obtains successful and significant achievements in the fields of computer vision and natural language processing (Strack, 2019; Zou et al., 2019; Tolkach et al., 2020). The capability of deep learning has been stronger than that of human in some certain fields. For example, the classification accuracy of ResNet is significantly higher than that of human on the ImageNet data set, and AlphaGo performs better than the human champion at playing chess (Singh et al., 2017; Lu et al., 2018). However, current machine learning algorithms depend highly on a large amount of labeled data. In some practical applications, the cost of data labeling is expensive. For example, it requires experienced doctors to spend a large amount of time to label the images in detail. Therefore, it is vital to investigate the few-shot learning method, which has higher generalization capability based on a small limited amount of labeled data. Using machine learning models, such as support vector machine (SVM) or convolution neural networks (CNNs), it is difficult to realize the few-shot learning capability because the lack of enough training data will cause the overfitting problem. SNN-based few-shot learning is a novel perspective for few-shot learning tasks, which is a promising approach to solve this kind of problem.

The learning capability of current SNN models still suffers from their robust adaptation to the environment with non-Gaussian noise, which severely limits the application of spike-driven models in real-world problems. Correntropy is a kind of non-linear local similarity measure in kernel space, which is closely related to the cross-information potential (CIP) in information-theoretic learning (ITL) (Chen et al., 2018). The main advantages of correntropy include two aspects. The first aspect is that it has the local property of providing an effective mechanism to weaken the influence of outliers and non-Gaussian noise. Another major advantage is that it introduces a novel measure method in sample space. If the samples are close to each other, the measurement is similar to the L2 norm. If the samples separate from each other, the measurement is similar to the L1 norm. When the samples are far away from each other, the measurement finally approaches the L0 norm. Due to its robustness to outliers and non-Gaussian noise, the correntropy theory has been widely applied in various fields, including signal processing and machine learning (Du et al., 2018; Luo et al. (2018), and Chen et al., 2019a).

In recent years, some novel entropy-based learning principles have been proposed for robust learning, such as the maximum mixture correntropy criterion (MMCC) (Wang et al., 2021). Previous studies have revealed that MMCC is a better selection than current optimization criteria, including the minimum mean square error (MMSE) criterion (Chen et al., 2019b). The MMSE criterion depends on the assumption that the data are noise-free or obey the Gaussian distribution. Once the assumption is not satisfied, such as the data disturbed by heavy-tailed noise, the performance of current machine learning algorithms may be severely reduced. Therefore, this work proposes to adopt the MMCC as the optimization criterion to rederive a novel spike-driven few-shot online learning (SFOL) model, resulting in a heterogeneous ensemble-based SFOL (HESFOL). The proposed model can perform robust few-shot online learning for sequential data. The paper is organized as follows: Section “Introduction” describes the preliminaries of this study, including SNN and entropy-based learning theory. The proposed HESFOL model is introduced and explained in Section “Materials and Methods.” Section “Results” presents the experimental results. And finally, the discussions and conclusions are proposed in Sections “Discussion” and “Conclusion,” respectively.

## Background

This study focuses on the two major broad areas of research, which are few-shot learning based on meta-learning method, and the entropy-based methods for machine learning. In this section, the related work in these two fields are covered and summarized.

### Few-Shot Learning Model Based on a Meta-Learning Framework

Few-shot learning based on the meta-learning method majorly uses the idea of learning-to-learn to realize the ambition. For example, meta-learning with augmented memory neural networks can solve the problem of how to quickly encode the vital information of new tasks by introducing an additional memory module (Santoro et al., 2016; Wang Y. et al., 2020). Model-agnostic meta-learning aims to learn a good initialization for the model, so that it can achieve good classification performance with only one or several gradient updates when facing a new task. Specifically, MAML introduces a new gradient, i.e., the two-order gradient, to find the most sensitive direction of the gradient change for fast learning of the new task. Gidaris et al. (2019) simultaneously identified the training category and the new category, and presented a dynamic network to generate the corresponding classification weight for the new category by designing a weight generator (meta-learner) based on the attention mechanism. Sun et al. (2019) presented a meta-transfer learning method, which pre-trains a feature extractor on the auxiliary data set and then fine tunes a learner based on a small amount of training data from the new tasks. Although there are a series of previous works to solve the few-shot learning problem using the meta-learner, there is no effective work based on SNN model to realize the few-shot learning performance by combining the brain mechanism with the machine learning theory, such as entropy learning theory.

### Information-Theoretic Learning

The information-theoretic learning approach has been widely applied to improve the performance of machine learning algorithms in recent years. Zadeh and Schmid (2020) presented an alternative loss derived from a negative log-likelihood loss that results in much better calibrated prediction rules. Zhang et al. (2020) presented to learn saliency prediction from a single noisy labeling based on entropy theory. To optimize the performance of current learning algorithms, researchers have focused on the correntropy-based method. Zheng et al. (2020) presented a mixture correntropy-based kernel-based extreme learning machine (MC-KELM) to improve the robustness of KELM, which adopts the recently proposed MMCC as the optimization criterion, instead of using the MMSE criterion. Heravi and Hodtani (2018) presented a group of novel robust information theoretic backpropagation (BP) methods, such as correntropy-based conjugate gradient BP (CCG-BP). Xing et al. (2019) presented a novel correntropy-based multiview subspace clustering (CMVSC) method to efficiently learn the structure of the representation matrix from each view and make use of the extra information embedded in multiple views. Ensemble algorithms can also be used for improving the robustness of learning tasks, such as clustering. Bootstrap AGGregratING (Bagging) algorithms were proposed to improve the classification by combining the classification of randomly generated data sets (Fischer and Buhmann, 2003). Bagging is a successful example of an independent ensemble classifier to train the model independently and then combine the outputs for the final verdict. Although there are a number of studies on correntropy-based machine learning, there still lacks an efficient and effective way to adopt the entropy theory in the application of spike-based machine learning. Therefore, this study aims at presenting an optimized entropy-based spike-driven few-shot learning with ensemble loss functions for robust few-shot learning.

## Materials And Methods

### Proposed Ensemble Loss

In this study, a novel objective function is proposed, which is the combination of single losses and integrates the proposed objective function into the spike-driven few-shot learning model. First, a mathematical explanation of the meaning of the proposed loss function is given to clarify the importance of the loss function. Let *ŷ* represents the estimated label of a true label *ŷ*. A loss function *L*(*y*, *ŷ*) represents a positive function, which indicates the difference between *ŷ* and *y*. Several types of loss functions are combined with trainable weights. Let ${\left\{L_{j}\left(y,\hat{y}\right)\right\}}_{j=1}^{K}$ represents *K* single loss functions. The aim is to find the best weights {λ_1_, λ_2_,., λ*_*K*_*} to combine *K* basis loss function for the generation of the best application-oriented loss function. A further constraint is added to avoid values close to 0 for all the weights. The proposed ensemble loss function is expressed as


(1) $$L=\sum_{i=1}^{K}\lambda_{i}L_{i}\left(y,\hat{y}\right),\sum_{i=1}^{K}\lambda_{i}=1.$$

The optimization with *N* training samples can be expressed as


(2) $$\begin{array}{c} \underset{w,\lambda }{\text{minimize}}\sum_{i=1}^{N}\sum_{j=1}^{N}\lambda_{j}^{2}L_{j}\left(y_{i},{\hat{y}}_{i}\right)\\ s.t.\sum_{j=1}^{K}\lambda_{j}^{2}=1 \end{array}.$$

Then, the constraint is incorporated as a regularization term according to the concept of Augmented Lagrangian. The modified objective function based on Augmented Lagrangian is described as


(3) $$\begin{array}{c} \underset{w,\lambda }{\text{minimize}}\sum_{i=1}^{N}\sum_{j=1}^{N}\lambda_{j}^{2}L_{j}\left(y_{i},{\hat{y}}_{i}\right)+\eta_{1}\left(\sum_{j=1}^{K}\lambda_{j}^{2}-1\right)+\\ \eta_{2}{\left(\sum_{j=1}^{K}\lambda_{j}^{2}-1\right)}^{2}. \end{array}$$

First and second terms of the objective function induce the values of $\lambda_{i}^{2}$ to approach 0 but the third term satisfied $\sum_{j=1}^{K}\lambda_{j}^{2}=1$. The overall training process is described in Algorithm 1.

**Algorithm 1: Pseudo-code of the whole training process for the proposed method.**

**Table:** 

**Input:**
The training set *T*, parameters *λ_*i*_* (Weights associated with each loss function), η_1_, η_2_ (Lagrangian weights), σ (Correntropy kernel bandwith), and *m* [maximum number of iterations (epochs)]
Base loss functions {*L*_*j*_(*X*_*i*_,*y*_*i*_)}^4^*_*j*_*_=1_, *K* = 4 (MMCC, Cross-entropy, MMSE based on firing rate, MMSE based on membrane potential)
**Output:**
Parameter *W*, λ_1_, λ_2_, λ_3_, λ _4_
1: Initiate Ensemble Loss Function using {*L*_*j*_(*X*_*i*_,*y*_*i*_)}^4^*_*j*_*_=1_ and random λ_1_, λ_2_, λ_3_, λ_4_
2: Initialize parameters *W*~*N*(0,Σ) and *t* = 0
3: **while** not converged **do**
4: Select a mini-batch of training samples {*X*_*i*_, *y*_*i*_}*_*i*_*_=1_*^N^* from training set *T*.
5: Perform a forward path, calculate the loss and regularization term:
$\sum_{i=1}^{N}$$\sum_{j=1}^{K=4}$$\lambda_{j}^{2}$*L*_*j*_(*y*_*i*_, *ŷ_*i*_*) + η_*1*_($\sum_{j=1}^{K=4}$$\lambda_{j}^{2}$−1) + η($\sum_{j=1}^{K=4}$$\lambda_{j}^{2}$−1)
6: Perform a backward propagation by the BPTT algorithm
7: Update W,λ_1_, λ_2_, λ_3_, λ_4_ by gradient descent algorithm.
8: *t*←*t*+1
**return** {*W*(*t*), λ_1_(*t*), λ_2_(*t*), λ_3_(*t*), λ_4_(*t*)}

### Mixture Maximum Correntropy Criterion

The correntropy has been widely used in various kinds of fields, such as machine learning and signal processing, which is defined as


(4) $$V_{\sigma }\left(X,Y\right)=E\left[k_{\sigma }\left(X,Y\right)\right]=\int \int k_{\sigma }\left(x,y\right)f_{XY}\left(x,y\right)dxdy$$

where *X* and *Y* represent the stochastic variables, and *E*[.] represents the expectation operator. The function *k*_σ_ (.,.) represents the kernel function with kernel width σ, and *f*_*XY*_(.,.) represents the joint probability density function (PDF). In practical engineering projects, PDF is usually unknown. Therefore, the sample estimator can be defined by finite usable samples as


(5) $${\hat{V}}_{\sigma }\left(X,Y\right)=\frac{1}{N}\sum_{i=1}^{N}k_{\sigma }\left(x_{i}-y_{i}\right).$$

The radial basis function is usually selected as the function of correntropy, which can be formulated as


(6) $${\hat{V}}_{\sigma }\left(X,Y\right)=\frac{1}{N}\sum_{i=1}^{N}k_{\sigma }\left(x_{i}-y_{i}\right)=\frac{1}{N}\sum_{i=1}^{N}\exp\left(-\frac{{||x_{i}-y_{i}||}^{2}}{2\sigma^{2}}\right).$$

As a local similarity measurement, the correntropy can effectively inhibit the influence of the outlier and the non-Gaussian distribution. Only if the variables *X* = *Y*, the correntropy reaches the maximum value, which is defined as maximum correntropy criterion (MCC). It can be used as the optimization criterion and robust loss function.

Therefore, this study uses a mixture correntropy, which can be described as


(7) $$V_{\sigma }\left(X,Y\right)=E\left[\sum_{s=1}^{S}\lambda_{s}G_{\sigma s}\left(X,Y\right)\right].$$

where ${\left\{G_{\sigma s}\left(.,.\right)\right\}}_{s=1}^{S}$ are *S* different Gaussian kernels based on each kernel size σ*_*s*_*. ${\left\{\lambda_{s}\right\}}_{s=1}^{S}$ are *S* mixture parameters satisfying 0 ≤ λ*_*s*_* ≤ 1 and $\sum_{s=1}^{S}\lambda_{s}=1$. In this paper, *S* is selected to be 2. Thus, the sample estimator of mixture correntropy can be expressed as


(8) $$\begin{array}{c} {\hat{V}}_{\sigma }\left(X,Y\right)=\frac{1}{N}\sum_{i=1}^{N}\left[\lambda G_{\sigma 1}\left(x_{j},y_{j}\right)+\left(1-\lambda \right)G_{\sigma 2}\left(x_{j},y_{j}\right)\right]\\ =\frac{1}{N}\sum_{i=1}^{N}\left[\lambda \exp\left(-\frac{{||x_{i}-y_{i}||}^{2}}{2\sigma_{1}^{2}}\right)+\left(1-\lambda \right)\exp\left(-\frac{{||x_{i}-y_{i}||}^{2}}{2\sigma_{2}^{2}}\right)\right] \end{array}.$$

**FIGURE 1:**
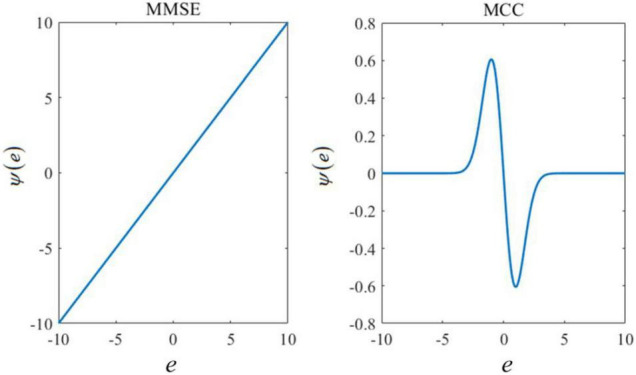
Influence functions based on the minimum mean square error (MMSE) or maximum correntropy criterion (MCC).


An unknown parameter can be estimated by maximizing the mixture correntropy between the desired signals and the estimated values. More details on the MMCC can be found in Zheng et al. (2020). The curve of influence functions of MCC and MMSE are shown in Figure 1. In this figure, the *x*-axis *e* represents the estimated error between the actual output and its corresponding estimate. The influence function Ψ(*e*) is calculated as follows:

(9) $$\Psi \left(e\right)=\frac{\partial G_{\sigma }\left(e\right)}{\partial e}=-\frac{e}{\sigma^{2}}\exp\left(-\frac{e^{2}}{2\sigma^{2}}\right)$$

where *G*_σ_ (⋅) represents the Gaussian kernel and σ is the size of the Gaussian kernel. It is shown that the influence function of MMSE increases linearly with the amplitude of the estimated error, while MCC is constrained to larger errors. Since larger errors are induced by outliers, MCC is useful to deal with the robust learning problem.

### Cross-Entropy Loss Function

The cross-entropy loss function is also regarded as log loss and is the most commonly used loss function for back propagation. It also increases as the predicted probability deviates from the actual label, which can be expressed as follows:


(10) $$L_{ce}\left({\hat{y}}_{i},y_{i}\right)=-\sum_{i}y_{i}\log\left({\hat{y}}_{i}\right).$$

In this study, a label *l^n^* is used for each image, which assumes a value of 1 only for images that belong to the same class as the image in the test phase and assumes a value of 0 otherwise. Then, the formulation can be described as


(11) $$E_{C}=\sum_{n=1}^{5}-l^{n}\log\sigma \left(y^{20+20\cdot n}\right)-\left(1-l^{n}\right)\log\left(1-\sigma^{20+20\cdot n}\right)$$

where the output of the SNN model only counts after all the images are fully presented.

### Regularization by Minimum Mean Square Error

To obtain a sparse firing regime, additional terms are added for the regularization of spiking activities. Two types of regularization methods are employed, including firing rate regularization and voltage range regularization. Firstly, to keep the average firing rate *f*_*j*_ for all neurons *j* close to a predefined target firing rate *f*_*target*_, a term is added, which is defined as


(12) $$\lambda_{f}E_{rate}=\lambda_{f}\sum_{j}{\left(f_{j}-f_{\text{target}}\right)}^{2}$$

where *f*_*j*_ is computed as the average spike count, which is expressed as


(13) $$f_{j}=\frac{1}{N_{batch}T}\sum_{n=1}^{N_{batch}}\sum_{t=1}^{T}z_{j}^{\left(n,t\right)}$$

where $z_{j}^{\left(n,t\right)}$ indicates the neural spikes in a particular batch with *n*, and *T* represents the total duration on a particular task. In addition, the factor λ*_*f*_* represents a hyperparameter that scales the importance of firing rate regularization.

Besides, to encourage the membrane potential to remain in a particular range, the membrane potential values are penalized, which are defined as


(14) $$\begin{array}{c} {\hat{V}}_{R}\left(v_{j}^{\left(n,t\right)},A_{j}^{\left(n,t\right)}\right)=\frac{\lambda_{v}}{NT}\sum_{i=1}^{N}\sum_{t=1}^{T}\sum_{j}(\max{\left(0,v_{j}^{\left(n,t\right)}-A_{j}^{\left(n,t\right)}\right)}^{2}\\ +\max{\left(0,-v_{j}^{\left(n,t\right)}-v_{th}\right)}^{2}) \end{array}$$

where an index *n* is used to indicate each batch. The variables *v_*j*_*^t^** and *a_*j*_*^t^** represent the membrane potential and the adaptive firing threshold, respectively. The resultant threshold voltage is *A*_*j*_(*t*). The factor λ*_*v*_* represents a hyperparameter that scales the importance of the resulting membrane potential regularization.

### Network Architecture of the Proposed Heterogeneous Ensemble-Based Spike-Driven Few-Shot Online Learning Model

**FIGURE 2:**
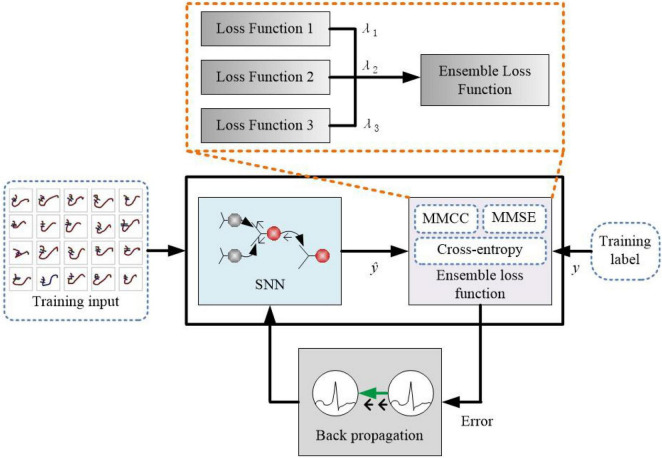
Schematic block figure of the proposed heterogeneous ensemble-based spike-driven few-shot learning (HESFOL) model.

In this study, the proposed HESFOL model contains a SFOL model with spiking neurons along with the ensemble loss function for back propagation. The proposed learning method is shown in Figure 2, where the ensemble loss function is represented by the dashed box. The combination of the loss function is based on Equations (1)–(3), which contains MMCC, cross-entropy loss function, and the two types of MMSE. Assume that in a multi-class data set *X*, *x*_*i*_∈*R^K^* represents the *k*-dimensional input. *y*∈{0,1}*^C^* represents the one-shot encoding of the label. Figure 2 depicts the proposed HESFOL model, extended with our ensemble loss function for the few-shot learning problem. In the backward step, the gradients of the proposed loss function flow back through the networks and weights. The weights are updated in the opposite direction of the gradient because the weights are determined and adjusted to decrease the loss value.

### Two-Compartment Spiking Neuron Model With Adaptation Mechanism

This study uses a two-compartment spiking neuron model for robust learning. Previous research has demonstrated that spike-driven learning with dendritic processing can fasten the convergence speed and reduce the number of spikes (Yang et al., 2021a). Therefore, a spiking and dendrite neuron model is proposed in this study. The soma compartment has two variables, which are the membrane potential *v_*j*_*^t^** and the adaptive firing threshold *a_*j*_*^t^**. The resulting threshold voltage *A*_*j*_(*t*) increase along with each output spike and decays to the baseline threshold *v*_*th*_ based on an adaptation time constant *τa*. Specifically, the soma compartment can be formulated as


(15) $$z_{j}\left(t\right)=H\left(v_{j}\left(t\right)-A_{j}\left(t\right)\right)$$

(16) $$A_{j}\left(t\right)=\lambda a_{j}\left(t\right)+v_{th}$$

(17) $$a_{j}\left(t\right)=\mu a_{j}\left(t-1\right)+z_{j}\left(t-1\right)$$

where μ = *e*^−Δ*t*/τ_*a*_^. The factor λ represents the impact of threshold adaptation. The discretion form of the spiking soma and dendrite models can be formulated as


(18) $$\begin{array}{c} \tau \frac{V_{i}\left(N+1\right)-V_{i}\left(N\right)}{\Delta T}=-V_{i}\left(N\right)+\frac{g_{b}}{g_{l}}\left(V_{i}^{b}\left(N\right)-V_{i}\left(N\right)\right)\\ +\sum_{i\neq j}W_{ji}^{rec}z_{i}\left(N-D\right) \end{array}$$

(19) $$V_{i}^{b}\left(N\right)=\sum_{j=1}^{m}W_{ij}s_{j}^{input}\left(N\right)+b_{i}$$

where *g*_*l*_ and *g*_*b*_ represent the leak conductance and the basal dendrite conductance, respectively, and Δ*T* represents the integration step. *W*_*ji*_*^rec^* represents the synaptic weight from the neuron *i* to the neuron *j* in the recurrent architecture, and D represents the transmission delay of recurrent spikes accordingly. The parameter τ = *C*_*m*_/*g*_*l*_ represents a time constant, where *C*_*m*_ represents the membrane capacitance. The variable *z*_*i*_ represents the output spikes of the *i*th spiking neuron. The variables *V*_*i*_ and *V_*i*_*^b^** represent the membrane potentials of soma and basal dendrite of the *i*th neuron, respectively. The term *W*_*ij*_ represents the synaptic weights in the input layer, and the constant *b*_*i*_ is defined as a bias term. The variable *s^input^* is calculated based on the following equation:


(20) $$s_{j}^{input}\left(t\right)=\sum_{k}\kappa \left(t-t_{jk}^{input}\right)$$

where *t*_*jk*_*^input^* represents the *k*th spiking time of the input neuron *j*, and the response kernel is expressed as follows:


(21) $$\kappa \left(t\right)=\left(e^{-t/\tau_{L}}-e^{-t/\tau_{s}}\right)\Theta \left(t\right)/\left(\tau_{L}-\tau_{s}\right)$$

where *τ_*L*_* and *τ_*s*_* represent long and short time constant, and Θ represents the Heaviside step function.

### Spike-Driven Online Learning Model

In the proposed HESFOL model, a regular leaky integrate-and-fire (LIF) neuron model is used, which is modeled based on the membrane potential *v*_*j*_(*t*) at time *t*. The membrane potential can integrate the input current and decay to a resting potential based on its membrane time constant *τ_*m*_*. Each time *v*_*j*_(*t*) reaches the threshold, the neuron generates a spike as *z*_*j*_(*t*) = 1. The regular spiking neuron model can be expressed as


(22) $$z_{j}\left(t\right)=H\left(v_{j}\left(t\right)-A_{j}\left(t\right)\right)$$

(23) $$A_{j}\left(t\right)=\lambda a_{j}\left(t\right)+v_{th}$$

where *W_*ji*_*^rec^** represents the synaptic weight from the neuron *i* to the neuron *j*, and *W_*ji*_*^in^** represents the weight of input component *x*_*i*_(*t*) for the neuron *j*. The factor describes the decay speed of the membrane potential, and *H* and *d* represent the Heaviside step function and the transmission delay of recurrent spikes, respectively. A refractory period *t*_*refrac*_ is used to set *z*_*j*_(*t*) = 0 after a neural spike. The outputs from the proposed HESFOL model are constructed by a weighted sum of low-pass filtered spikes, which is defined as


(24) $$y_{k}\left(t\right)=\left(1-\nu \right)\sum_{t^{\prime }\leq t}\sum_{j}\nu \left(t-t^{\prime }\right)W_{kj}^{out}z_{j}\left(t^{\prime }\right)+b_{k}^{out}$$

where *W*_*kj*_*^out^*, *b*_*k*_*^out^*, ν = *e*^−Δ*t*/τ_*out*_^, and τ_*out*_ are the readout time constants.

In the proposed HESFOL model, an associated eligibility trace is considered at each synapse, which is the key concept of the *e*-prop algorithm. The eligibility trace *e*_*ji*_(*t*) represents the influence of the weight *W*_*ji*_ on the spiking activities of the neuron *j* at time *t*, but requires taking into account dependencies that do not involve other neurons besides *i* and *j*. Eligibility traces exist separately for input and recurrent synapses. The variable *h*_*j*_(*t*) represents the hidden variables for a neuron *j* at time *t*. Then, the dynamics of the eligibility trace is defined as follows:


(25) $$e_{ji}\left(t\right)=\frac{\partial z_{j}\left(t\right)}{\partial h_{j}\left(t\right)}\cdot \varepsilon_{ji}\left(t\right)$$

(26) $$\varepsilon_{ji}\left(t\right)=\frac{\partial h_{j}\left(t\right)}{\partial h_{j}\left(t-1\right)}\cdot \varepsilon_{ji}\left(t-1\right)+\frac{\partial h_{j}\left(t\right)}{\partial W_{ji}}$$

The eligibility vector *ε_ji_*(*t*) means that the quantity is propagated forward in time along with the computation of the proposed HESFOL model. The term $\frac{\partial z_{j}\left(t\right)}{\partial h_{j}\left(t\right)}$ cannot be calculated directly because the relationship between *z*_*j*_(*t*) and *h*_*j*_(*t*) contains the non-differentiable Heaviside function. Therefore, the derivative in Equation (22) is replaced with a pseudo derivative that is described as


(27) $$\Psi_{j}\left(t\right)=0.3\cdot \max\left(0,1-\left|\frac{v_{th}-v_{j}\left(t\right)}{v_{th}}\right|\right).$$

The vector of hidden variables *h*_*j*_(*t*) is defined by *h*_*j*_(*t*) = *v*_*j*_(*t*), and the eligibility traces applied in the LIF dynamics can be formulated as


(28) $$e_{ji}\left(t\right)=\psi_{j}\left(t\right)\cdot {\bar{z}}_{i}\left(t-d\right)$$

where ${\bar{z}}_{i}\left(t\right)=\sum_{t^{\prime }\leq t}\alpha^{t-t^{\prime }}z_{i}^{t^{\prime }}$ is defined as the low-pass filtered presynaptic spiking activities of the neuron *i*. In addition, the vector of hidden variables of a neuron, *h*_*j*_(*t*), also contains the variable of the firing threshold *h*_*j*_(*t*) = [*v*_*j*_(*t*), *a*_*j*_(*t*)]. For the adaptive LIF (ALIF) neuron model, the eligibility trace *e*_*ji*_(*t*) is defined as


(29) $$e_{ji}\left(t\right)=\Psi_{j}\left(t\right)\left({\bar{z}}_{i}\left(t-d\right)-\beta \varepsilon_{a,ji}\left(t\right)\right),$$

(30) $$\begin{array}{c} \varepsilon_{a,ji}\left(t\right)=\left(\rho -\beta \cdot \Psi_{j}\left(t-1\right)\right)\varepsilon_{a,ji}\left(t-1\right)\\ +\Psi_{j}\left(t-1\right){\bar{z}}_{i}\left(t-d-1\right) \end{array}.$$

To realize the plasticity of the proposed HESFOL model, the derivative of the Heaviside function $\frac{\partial H\left(v_{j}\left(t\right)-v_{th}\right)}{\partial v_{j}\left(t\right)}$ is replaced with a pseudo derivative in the backward pass, which is formulated as


(31) $$\Psi_{j}\left(t\right)=0.3\cdot \max\left(0,1-\left|\frac{v_{th}-v_{j}\left(t\right)}{v_{th}}\right|\right).$$

In addition, the derivative of the Heaviside function $\frac{\partial H\left(v_{j}\left(t\right)-A_{j}\left(t\right)\right)}{\partial v_{j}\left(t\right)}$ is replaced by the formula as


(32) $$\Psi_{j}\left(t\right)=0.3\cdot \max\left(0,1-\left|\frac{A_{j}\left(t\right)-v_{j}\left(t\right)}{v_{th}}\right|\right)$$

where the actual update to the initial synaptic weight *W*_*init*_ of the proposed HESFOL model is realized by the application of Adam with a learning rate η_*rate*_.

## Results

### Details of the Heterogeneous Ensemble-Based Spike-Driven Few-Shot Online Learning Architecture

**FIGURE 3:**
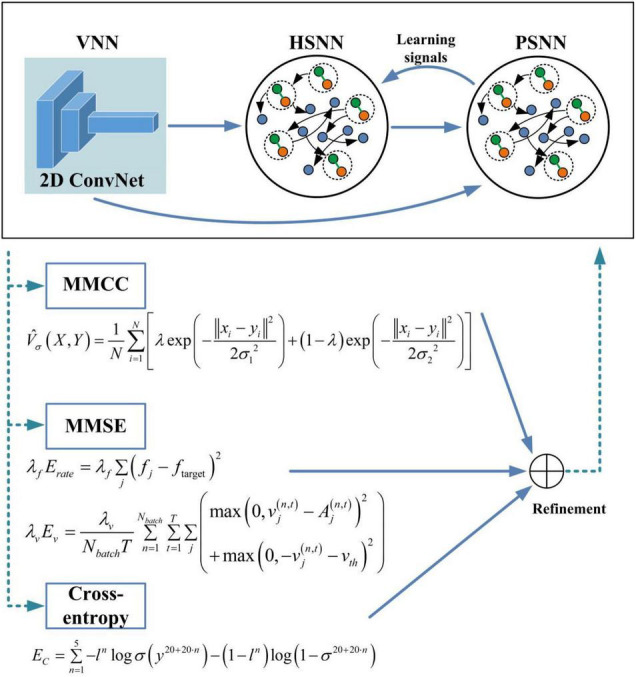
An overview of the proposed HESFOL framework. We employ 2D convolution for the ConvNet, which is considered as a visual-pathway-inspired neural network (VNN). In addition, two subnetworks are realized, which are hippocampus-inspired SNN (HSNN) and PFC-inspired SNN (PSNN). The learning signals are transmitted from PSNN to HSNN.

As shown in Figure 3, the overall architecture of the proposed HESFOL model contains two parts, which are the SFOL model and the ensemble loss. The SFOL model is inspired by the neural mechanism underlying the human brain, which is based on the interaction between the hippocampus and the prefrontal cortex (PFC). Therefore, there are two modules in the SFOL model, which are hippocampus-inspired SNN (HSNN) and the PFC-inspired SNN (PSNN). The external inputs are summed and integrated into the membrane potentials of neurons in HSNN and PSNN modules. The HSNN readout is composed of the weighted low-pass filtered spike trains of neurons in the HSNN module. Suppose there exists an infinitely large family *F* of possibly relevant learning tasks *C*. The HSNN module learns a particular tasks *C* from *F* based on the learning signals provided by the PSNN module. Each time HSNN receives the new C tasks from the family *F*, the synaptic weight is updated. The learning performance of HSNN on the task *C* is evaluated based on the loss function. After the first phase of learning, the parameters are fixed between HSNN and PSNN modules, and new *C* tasks from the family *F* are selected to evaluate the HSNN learning performance. The encoding module of the SFOL model uses the processing mechanism of the visual pathway, so there is a visual-pathway-inspired neural network (VNN) based on the 2D ConvNet. The images are input into the VNN in a pixel array manner for input encoding. The 2D ConvNet consists of three layers, which is based on the non-spiking McCulloch–Pitts neuron model. HSNN contains 180 two-compartment LIF (TLIF) neurons and 260 conventional LIF neurons. The learning signals can be only transmitted from PSNN to HSNN in the first phase. To realize the outer loop optimization, the ensemble loss is employed in the BPTT algorithm, which contains the loss functions of the MMCC, MMSE, and cross-entropy loss. The values of the hyperparameters used in the HESFOL model are listed in Table 1.

**TABLE 1:** Hyperparameter list used in the heterogeneous ensemble-based spike-driven few-shot online learning (HESFOL) architecture.

Parameters	Description	Values
τ_*m*_	Timing constant of membrane	15 ms
τ_*out*_	Timing constant of readout neurons	10 ms
*d*	Synaptic transmission delay	1 ms
*t*_*refrac*_	Refractory period duration	5 ms
*f*_*target*_	Target firing rate	20 Hz
η*_*out*_*	Learning rate of outer loop	2 × 10^–3^
λ*_*f*_*	Spike rate regularization	1.0
*v*_*th*_	Threshold	1.0
λ*_*v*_*	Voltage regularization	10^–2^
*t*_*img*_	Number of time steps per image	20 ms
*τ_*a*_*	Adaptation timing constant	200 ms
η	Learning rate	1.915 × 10^–3^
*N*_*HSNN*_	Network size of HSNN	447
*q*_*ada*_	Neuron fractions using adaptation	40.5%
β	Impact of threshold adaptation	0.4902
*N*_*batch*_	Batch size for outer loop optimization	285
*N*_*PSNN*_	Network size of the PSNN	239
*τ_*LS*_*	Timing constant learning signals of readouts	10 ms
*f*_*tarPSNN*_	Target firing rate for PSNN	20 Hz

### Few-Shot Learning Performance on Spike Patterns With Non-Gaussian Noise

In the first task, spiking patterns with the non-Gaussian noise are used to test the few-shot learning capability of the proposed HESFOL model. A spatiotemporal spike pattern classification task is considered, where each pattern is generated with the firing frequency ranging from 2 to 50 Hz. Indeed, the spike patterns describe the spatiotemporal dynamics of the neural population, in which the firing frequency and precise timing of spiking neurons contain the rich information of an external input of the environment. The spike patterns of each category are instantiated by adding the non-Gaussian noise to the corresponding template, which contains the Poisson noise and the spiking deletion noise. We first generate 1,000 spike pattern templates based on certain spiking neurons. Then, we generate 25 spike patterns for each template by randomly marking a uniform distribution of the neural firing rate. Therefore, we build a few-shot learning data set of the spike patterns with 1,000 classes and 25 samples for each class.

**FIGURE 4:**
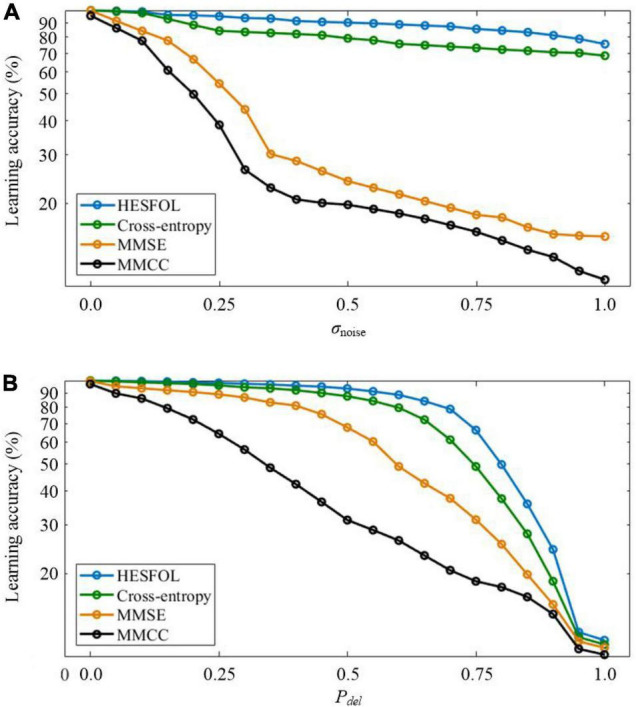
Comparison of the few-shot learning performance with non-Gaussian noise between the HESFOL model and the other models. **(A)** Few-shot learning accuracy with Poisson noise. **(B)** Few-shot learning accuracy with random deletion noise.

Two types of non-Gaussian noise are considered in few-shot learning in the spiking patterns classification task. In the first type, new noisy spatiotemporal pattern samples are generated by adding Poisson noise to the templates with the standard deviation (SD) of σ_*noise*_. In the second type, random deletion noise is added to the templates to generate new noisy spiking pattern samples, where each spike is randomly deleted according to a probability of *P*_*del*_. As shown in Figure 4, our proposed HESFOL model achieves remarkable performance in various noisy situations, highlighting the advantages of our heterogeneous ensemble-based approach. Among all the presented learning loss functions, the loss function with MMCC, MMSE, and cross-entropy loss is the best to realize the highest robustness to tolerate noise.

### Few-Shot Learning Performance With Non-Gaussian Noise

**FIGURE 5:**
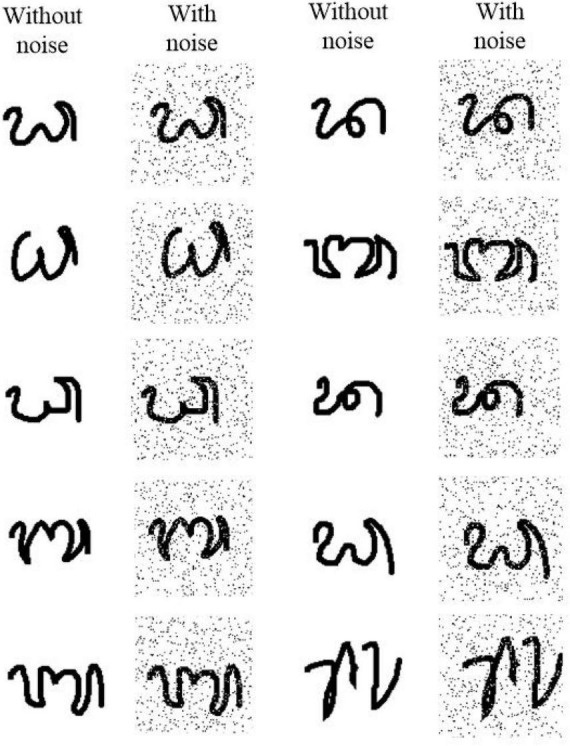
Images with non-Gaussian salt-and-pepper noise in the Omniglot data set using signal-noise rate of 1, 0.9, 0.7, and 0.5.

**FIGURE 6:**
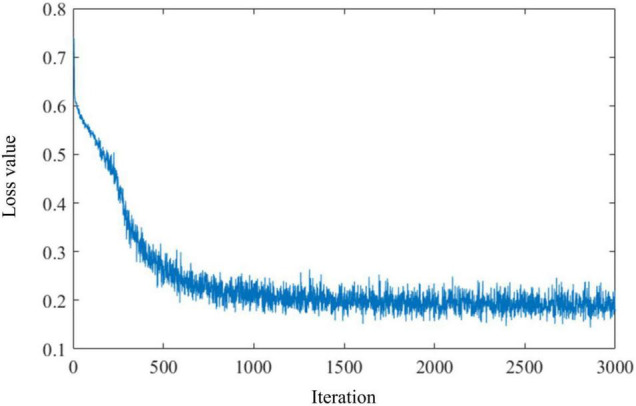
The evolution of the loss value based on the ensemble loss along with the iteration.

In this study, we test our HESFOL model using the Omniglot data set. The Omniglot data set contains a total of 1,623 classes and 32,460 images, and each class contains 20 images. The data set is split up into 964 training classes and 659 classes. There are two phases in the test, which means a sequence of images in which one image of the same class exactly appears in phase #2 as the one shown in phase #1. The 2D CNN with 15,488 neurons is organized into three layers, which contain 16, 32, and 64 filters, respectively. The kernel size used in the convolutional filters is 3 × 3. The average pooling layers and batch normalization layers are also used for optimization improvement in the HESFOL model. Salt-and-pepper noise is added to the Omniglot images by randomly flipping 15% of the images, which is a kind of non-Gaussian noise. Figure 5 shows the images in the Omniglot data set that are contaminated by the non-Gaussian salt-and-pepper noise. The loss value of the ensemble evolves with an iteration, which is shown in Figure 6. This reveals that the loss value of the proposed HESFOL model reduces to a stationary level of about 0.2 quickly within 1,000 iterations.

**FIGURE 7:**
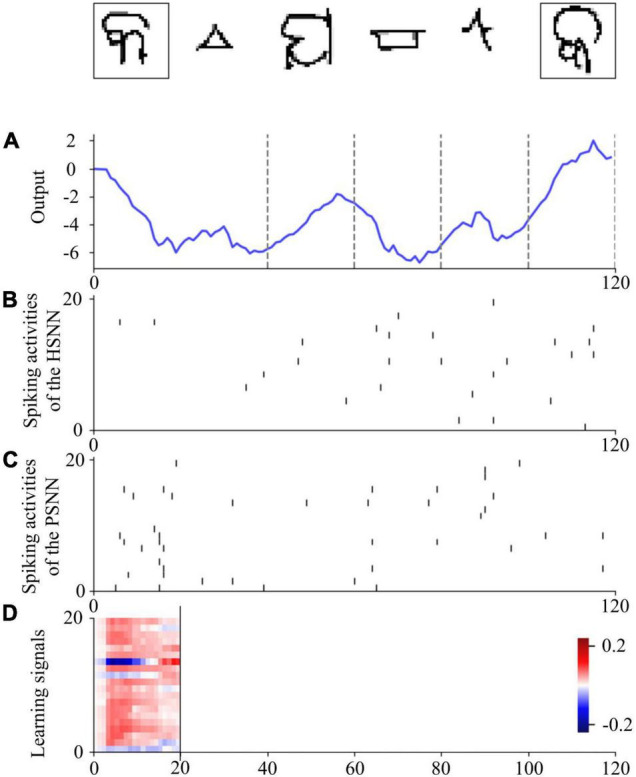
One sample trial for the few-shot learning on the Omniglot data set using the HESFOL model. **(A)** Output of the readout neuron. **(B)** Spiking activities of neurons in the HSNN module. **(C)** Spiking activities of neurons in the PSNN module. **(D)** Learning signals of PSNN for HSNN neurons.

The values 0 and 1 are used to encode phases #1 and #2, respectively, which are included in the input signal. Images from the Omniglot data set are presented to the VNN using the 28 × 28 grayscale pixels of arrays. A single output is used to determine in phase #2 whether the presented image belongs to the same class as that in phase #1. Spike-based learning is employed by the HESFOL model, and PSNN receives both the spiking activities from HSNN and the input information with phase ID. The learning signals are transmitted from PSNN to HSNN only in the first phase. Figure 7 shows the spiking activities of the proposed HESFOL model during the few-shot learning task on the Omniglot data set. This reveals that the sparse spiking activities of the HSNN and PSNN subsystems occur in the few-shot learning task. The ensemble loss, which contains MMCC, cross-entropy loss function, and two types of MMSE, can successfully solve the few-shot learning problem with the images with non-Gaussian noise.

### Few-Shot Learning Performance on Manipulator Control

**FIGURE 8:**
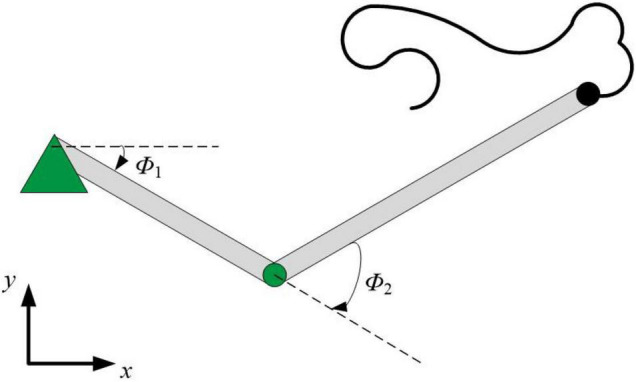
Few-shot motor control of the end-effector of a two-joint robotic arm.

**FIGURE 9:**
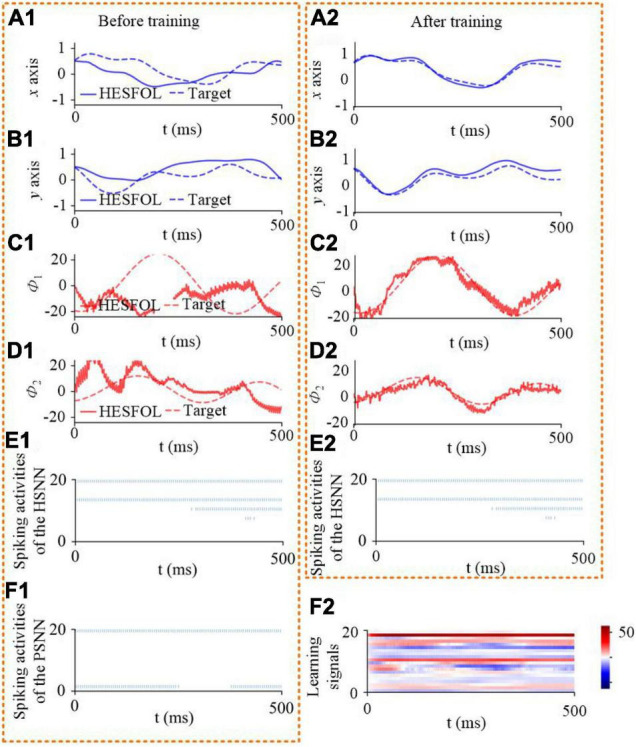
Few-shot motor control performance of the proposed HESFOL model. It shows the one-shot learning of a new end-effector movement in 500 ms. It reveals control performance and spiking activities before and after training. **(A1)** Position in the *x*-direction based on HESFOL control before training and the target position in the *x*-direction. **(A2)** Position in the *x*-direction based on HESFOL control after training and the target position in the *x*-direction. **(B1)** Position in the *y*-direction based on HESFOL control before training and the target position in the *y*-direction. **(B2)** Position in the *y*-direction based on HESFOL control after training and the target position in the *y*-direction. **(C1)** Motor command in the form of joint angular velocity and target angular velocity in the *x*-direction before training. **(C2)** Motor command in the form of joint angular velocity and target angular velocity in the *x*-direction after training. **(D1)** Motor command in the form of joint angular velocity and target angular velocity in the *y*-direction before training. **(D2)** Motor command in the form of joint angular velocity and target angular velocity in the *y*-direction after training. **(E1)** Spiking activities of HSNN before training. **(E2)** Spiking activities of HSNN after training. **(F1)** Spiking activities of PSNN. **(F2)** Learning signals generated by PSNN for HSNN.

**FIGURE 10:**
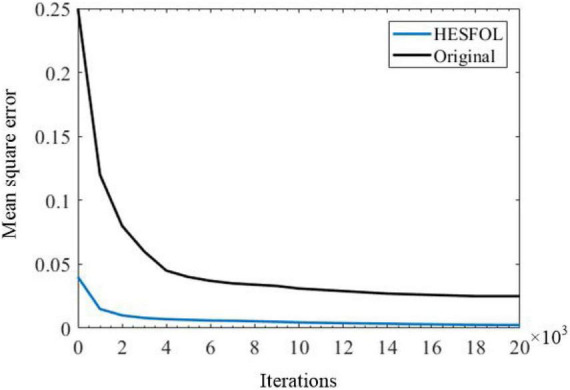
Control performance based on the mean square error of original and HESFOL models.

We further demonstrate the few-shot learning capability for manipulator control. The manipulator uses the end-effector of a two-joint arm for a generic motor control task to trace a target trajectory in Euclidean coordinates (*x*, *y*), as shown in Figure 8. In the motor control task, the proposed HESFOL model can learn to reproduce a particular randomly generated target movement with the actual movement of the arm end-effector. The learning task is divided into two trails, which contains a training and a testing trial. In the training trial, PSNN receives the target movement in Euclidean coordinates, and PSNN outputs the learning signals for the HSNN module. After the testing trial, the weight update is applied to HSNN. In the testing trial, HSNN is tested to reproduce the previously given target movement of the arm end-effector without receiving the target trajectory. The input of HSNN is the same across all trials and is given by a clock-like input signal. The output of HSNN is the motor commands for angular velocities of the joints ${\dot{\Phi }}^{t}=\left({\dot{\phi }}_{1}^{t},{\dot{\phi }}_{2}^{t}\right)$. As shown in Figure 9, the trajectory generated by HSNN as solid lines during both the training and testing trial. HSNN can regenerate the target movement based on biologically realistic sparse spiking activities after PSNN send learning signals to HSNN during the training trial. Figure 9 also shows the learning signals and the spiking activities of the proposed HESFOL model. The mean square error between the target and actual movement in the testing trial is shown in Figure 10. The result reveals that the HESFOL model with the ensemble loss performs better than the model with just one or less types of loss functions. This reveals that the proposed HESFOL provides a new point of view for efficient motor control and learning underlying the neural mechanism of the human brain.

### Effects of the Ensemble Parameters on Learning Performance

In this study, we further explore how each of the base loss functions in the ensemble loss of the proposed HESFOL model contribute to the ensemble loss function in Table 2. We test the effects of the ensemble parameters on the few-shot learning performance on different types of data sets, including spiking patterns and the Omniglot data set. Overall, the cross-entropy loss has the largest weights for both the data sets, which means that the cross-entropy contributes the most to form the ensemble loss function of the proposed HESFOL model.

**TABLE 2:** Test accuracies (%) of different ensemble parameter settings in the Omniglot data set.

Groups	Loss	Values	Omniglot accuracy	Groups	Loss	Values	Omniglot accuracy
Group 1	MMCC	0.1	90.6%	Group 5	MMCC	0.1	90.6%
	Cross	0.9			Cross	0.9	
	Rate	0.5			Rate	0.5	
	Vol	0.5			Vol	0.5	
Group 2	MMCC	0.1	90.6%	**Group 6**	MMCC	**0.2**	**93.1%**
	Cross	1.3			**Cross**	**0.8**	
	Rate	0.3			**Rate**	**0.5**	
	Vol	0.3			**Vol**	**0.5**	
Group 3	MMCC	0.1	92.2%	Group 7	MMCC	0.2	90.6%
	Cross	1.0			Cross	1.3	
	Rate	0.45			Rate	0.25	
	Vol	0.45			Vol	0.25	
Group 4	MMCC	0.1	91.4%	Group 8	MMCC	0.2	89.8%
	Cross	0.7			Cross	0.6	
	Rate	0.6			Rate	0.6	
	Vol	0.6			Vol	0.6	

*The bolded values are the optimal configuration.*

In terms of the correntropy loss function, the weight value of 0.1 tends to be a suitable loss function in a very noisy environment, especially in the presence of outliers. The proposed SNN architecture realizes the few-shot learning tasks by back propagating the gradient of the loss and it is likely to suffer from the problem of gradient vanishing. Thus, a loss function that highlights the error can outperform the MMCC loss function. Therefore, the weight of the cross-entropy loss function is larger than the others in the ensemble loss function of the proposed HESFOL model.

### Comparison With the Other Models on Few-Shot Learning Performance

To evaluate the few-shot learning performance more directly, we compare the HESFOL model with other models, including ANNs and SNNs. Jiang et al. (2021) proposed a novel SNN model with a long short-term memory (LSTM) unit for few-shot learning, called the multi-timescale optimization (MTSO) model. As the proposed HESFOL model has not used model augmentation to achieve the best accuracy, a fair comparison is conducted with the other models without augmentation and fine tuning. The MTSO model without augmentation can achieve 95.8% accuracy. In terms of ANN models, the MANN presented by Santoro et al. (2016) achieved 82.8% accuracy on the Omniglot data set. The learning accuracy of CNN presented by Jiang et al. (2021) only reached 92.1%, while the spiking CNN with L1 regularization for sparsity obtained 92.8% learning accuracy on Omniglot. The Siamese Net can get 96.7% accuracy with augmentation (Koch et al., 2015). The proposed HESFOL model achieved 93.1% accuracy on the Omniglot data set with non-Gaussian noise, which shows a comparative performance on the few-shot learning task. Although its learning accuracy is slightly lower than that of the Siamese Net, the HESFOL model uses a spike-based paradigm, which means that it owns the advantage of low power consumption and high biological plausibility. In addition, the HESFOL model is 2.7% lower than the MTSO, but the HESFOL model uses non-Gaussian noisy data to evaluate, other than the pure data set used by the MTSO model. This demonstrates that the proposed HESFOL model can achieve high robustness of few-shot learning without losing much accuracy. As the proposed HESFOL uses a simple spike-based few-shot learning framework, more complicated data set is not the aim of this study. However, we will conduct on more complicated data set in the future work. It should be noted that the major ambition is to present a robust spike-based few-shot learning framework based on the ITL theory.

### Effects of the Critical Parameters of the Heterogeneous Ensemble-Based Spike-Driven Few-Shot Online Learning Model on Learning Performance

**FIGURE 11:**
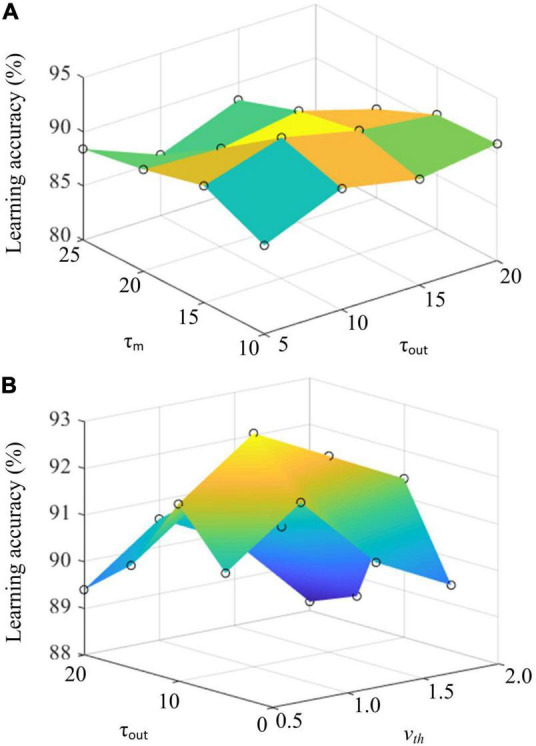
**(A)** The effects of timing constant of membrane τm and timing constant of readout neurons τout on learning accuracy. **(B)** The effects of membrane potential threshold vth and timing constant of readout neuorns τout on learning accuracy.

In addition, we further explore the critical parameter of the proposed HESFOL model on the few-shot learning performance. Three critical parameters are selected, which are the timing constant of membrane τ_*m*_, timing constant of readout neurons τ_*out*_, and membrane potential threshold *v*_*th*_. We select the Omniglot data set to test the learning performance of the HESFOL model. As shown in Figure 11, learning accuracy is demonstrated by changing parameters. Figure 11A reveals that the highest learning accuracy can be obtained when τ_*m*_ = 15 and τ_*out*_ = 1 0. In addition, Figure 11B shows that τ_*out*_ = 10 and *v_*th*_* = 1.0 can result in the highest learning accuracy. It also suggests the preferred parameter values for neural dynamics when realizing the classification tasks to test the few-shot learning performance. As the proposed HESFOL model realizes the few-shot learning capability based on the meta-learning scheme, it also implies that the SNN model with this set of parameter values has the highest LSTM performance to store *a priori* experience for the current learning task.

**FIGURE 12:**
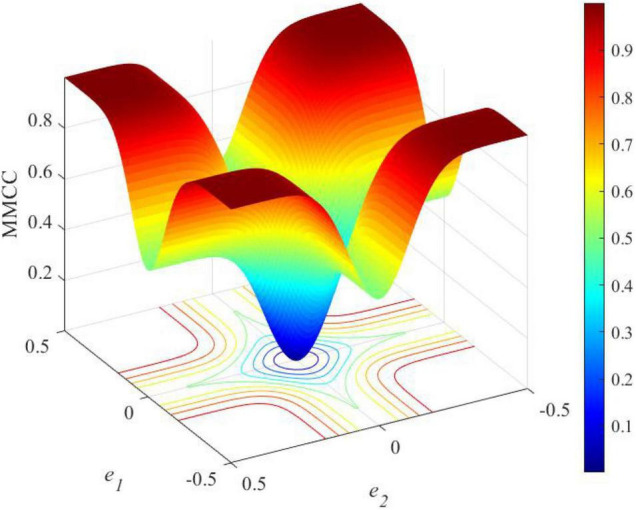
The loss function curve of MMCC along with the errors.

## Discussion

### Theoretical Analysis

The major components of a learning model are the loss function, which demonstrates the influence of samples on the model training. The loss function gives each sample a value, which demonstrates the participation level of each sample in the learning problem. For example, if the loss function assigns an outlier sample a large value, this outlier may generate a negative impact on the model parameters. If the 0–1 loss function penalizes all samples that are classified incorrectly with the value 1, this can be considered as robustness. A robust learning machine requires that outliers do not influence the system performance too much. The ultimate goal of a learning approach is to own the capability to classify unseen data. Therefore, the classifier should have robustness to data disturbance. A more difficult situation exists in the noisy environment, where the outlier will damage the training or testing data. To deal with the noisy environment, an efficient approach is to use a robust loss function. If there exists a constant *k* and samples with *e_*i*_* > *k* do not be set with a large value by the loss function, where *e*_*i*_ represents the error of the *i*th sample, this loss function can be regarded as robust. Despite some learning classifiers can classify the training data with high performance, it cannot estimate the unknown data. Therefore, although the training error is low, it will induce high generation error. This failure is due to the overfitting problem, which means that the classifier matches the training data and loses the generalization capability. A better generalization solution is to use a loss function to realize a more general classifier.

If an error value is expected to be minimized, the loss function will generate a more generalized classifier with an enhanced margin. If a classifier has an enhanced margin, the performance will be improved to deal with unseen data with better generalization. An enhanced classifier can be realized when the correct samples close to the classification line are penalized, and the loss function can be regarded as margin enhancing. As each loss function has its own advantages and disadvantages, there is no comprehensive loss function to work well in all situations. Therefore, this research proposes the use of an ensemble of loss in the SNN model. As correntropy is a bounded function, it is less sensitive to outliers. The kernel size limit the influence of each independent sample on the total result, which can reduce the effects of non-Gaussian noise in the environment on learning performance. Figure 12 further presents the loss function of MMCC. It shows that MMCC is a measure to evaluate the local similarity of samples and present a unique mixed norm feature, which is specifically summarized as follows:

1. MMCC shows the characteristics of the ℒ2 norm when the error is close to 0;
2. The MMCC loss function shows the characteristics of the ℒ1 norm when the error increases from 0;
3. The MMCC loss function demonstrates the characteristics of the ℒ0 norm when the error is particularly large.

Therefore, MMCC is sensitive to elements with high local similarity in the sample, but not to the two elements with large difference. Due to these characteristics, MMCC can effectively reduce the impact the non-Gaussian noise on learning tasks, inducing more robust spike-driven few-shot learning performance.

In addition, spiked dendrites in the HESFOL model also enhance the robustness of few-shot learning. It has been proven in some previous studies (Yang et al., 2021a). This is because the non-linear computation of spiked dendrites can inhibit the disturbance of input noise and in the transmission pathway, thus improving the learning performance. In addition, as spiked dendrites can solve the credit assignment problem and distinguish the information flow in feedforward and recurrent pathways, the learning performance, including robustness, can be further enhanced.

### Power Efficiency Based on the Heterogeneous Ensemble-Based Spike-Driven Few-Shot Online Learning Model

Previous research has revealed that the lowest energy consumption of a synaptic operation is about 20 pJ in the state-of-the-art neuromorphic system (Merolla et al., 2014; Qiao et al., 2015). The proposed HESFOL model will cost around 60 spikes in HSNN and around 70 spikes in PSNN on the classification task using the Omniglot data set. Therefore, single spike classification using the proposed HESFOL will cost 2.6 pJ in such a neuromorphic system, which outperforms the current work based on digital neuromorphic hardware (≈2 μJ) (Esser et al., 2016) and potentially 50,000 more power efficient than current graphics processing unit (GPU) platforms (Rodrigues et al., 2018). Our previous work has shown that the classification task using an improved DEP-based SNN model induces about 1,011 SynOps to obtain the highest classification accuracy (Yang et al., 2021a). Therefore, the proposed HESFOL model can reduce 87.14% of the totally induced spikes, i.e., the power consumption, in comparison with the state-of-the-art SNN model. The reasons for the low-power consumption by the proposed HESFOL model can be divided into three aspects. Firstly, the ensemble entropy theory is used, which can fasten the learning speed to reach the maximum learning accuracy. It is useful to reduce the power consumption cost during learning. Secondly, a few-shot learning procedure is used in the classification task, which will shorten the overall learning process and potentially reduce power consumption. Thirdly, spiked dendrites are used in the spike-driven learning task, which can further cut down the required spikes due to their non-linear information processing capability. Therefore, the proposed HESFOL model cannot only improve the learning accuracy and robustness of SNN models, but also further cut down the power efficiency of neuromorphic hardware.

### Comparison With Spiking Neural Networks of Liquid State Machines and Future Work

Previously, Roy et al. (2019) presented a good overview of recent SNN training techniques in the context of reservoirs or liquid state machines (LSMs) whose architectures are similar to the proposed HESFOL framework. LSMs use unstructured, randomly connected recurrent networks paired with a simple linear readout. As shown in Table 3, such frameworks with spiking dynamics have shown a surprising degree of success for a variety of sequential recognition tasks (Panda and Roy, 2017; Soures and Kudithipudi, 2019; Wijesinghe et al., 2019). Soures and Kudithipudi (2019) presented a deep LSM with an STDP learning rule for video activity recognition. Wijesinghe et al. (2019) presented the ensemble approach for LSM to enhance class discrimination, leading to better accuracy in speech and image recognition tasks compared to a single large liquid. Wang et al. (2020) proposed a novel LSM model for sitting posture recognition. Luo S. et al. (2018) presented two different methods to improve LSM for real-time pattern classification from the perspectives of spatial integration and temporal integration. We introduce LSM as a model for an automatic feature extraction and prediction from raw electroencephalography (EEG) with a potential extension to a wider range of applications. Al Zoubi et al. (2018) introduced LSM as a model for an automatic feature extraction and prediction from raw EEG with a potential extension to a wider range of applications. Although these works presented different strategies for sequential recognition tasks, none of them have successfully solved the few-shot learning problem. This study firstly proposed a unified framework for the simultaneous realization of robust image classification and few-shot learning performance, which is superior to representative LSM models based on the recurrent architecture.

**TABLE 3:** Comparison with the representative liquid state machine (LSM) models with the recurrent architecture.

Research	Application	Robustness	Few-shot learning
Soures and Kudithipudi, 2019	Video activity recognition	No	No
Wijesinghe et al., 2019	Image/speech recognition	No	No
Wang et al., 2020	Sitting posture recognition	No	No
Luo et al. 2018	Pattern classification	No	No
Al Zoubi et al., 2018	Emotion recognition	No	No
Panda and Roy, 2017	Visual recognition	Yes	No
HESFOL	Image classification	Yes	Yes

For deep SNN training, the ANN–SNN conversion requires less GPU computing than supervised training with surrogate gradients. Meanwhile, it has yielded the best performance on large-scale networks and data sets among the methodologies. For example, Ding et al. (2021) proposed a rate-norm layer to replace the ReLU activation function in source ANN training, enabling direct conversion from a trained ANN to an SNN. Zheng H. et al. (2020) also proposed a threshold-dependent batch normalization (tdBN) method based on the emerging spatiotemporal BP, enabling direct training of a very deep SNN and efficient implementation of its inference in neuromorphic hardware. These works have successfully realized pattern recognition functions on more complicated data set than the data set used in this research, and have achieved high performance on these tasks, such as classification on dynamic vision sensor- (DVS-) CIFAR10. However, none of these research have solved the few-shot learning problems, and learning robustness is also not focused and referred in these studies. In contrast, the proposed HESFOL model presented a robust few-shot learning framework with ITL approach, which is meaningful for combining the machine learning approach with brain-inspired SNN paradigms. On the other hand, future work will try to apply the ANN–SNN conversion technique in few-shot learning algorithms based on ANN models, and it will be further combined with the ITL method that is used and plays a major part in the robust few-shot learning performance of the HESFOL model.

One of the critical issues is to present efficient training algorithms for SNN models to deal with complicated data set for more realistic applications. Shallow SNNs can be trained based on surrogate gradient descent, but they can only achieve high performance on simple data sets, such as MNIST. In fact, the discrepancy between a forward spike activation function and a backward surrogate gradient function during training limits the learning capability of deep SNNs. There are a series of studies in which SNN has shown to be trained from scratch using the surrogate gradient descent approach. For example, Kim and Panda (2020) proposed a technique called Batchnorm through time (BNTT) for training SNNs that dynamically changes the parameters and has an implicit effect as a dynamic threshold. They also proposed a spike activation lift training approach, which is essentially a threshold fine-tuning or initialization step before the actual training (Kim et al., 2021a,b). These two models can train SNN models with deep layers, and they are tested on complicated data sets, such as DVS, CIFAR100, and Tiny ImageNet. They demonstrate high performance on deep SNN models, which can be scaled for more realistic application. Therefore, in the next step, the proposed HESFOL model will be combined with the BNTT algorithm for deep network training. For example, the proposed ITL approach will be added to the current BNTT framework to explore the learning robustness or efficiency, and the HESFOL model can be used in the modeling of a single layer in a deep SNN architecture. Thanks to the spiking dendrites of the HESFOL model, it can naturally solve the credit assignment problem between feedforward and feedback pathways. It is meaningful for application in more complicated tasks and practical situations.

Another future work is to apply the proposed HESFOL model in tasks beyond recognition experiments. Previous research has presented a series of possibilities for SNNs to target complicated tasks other than visual recognition. For example, Kim and Panda (2021) presented a visual explanation technique to analyze and explain the internal spiking behavior of deep temporal SNNs to make SNNs ubiquitous. Kim et al. (2021) explored the applications of SNN beyond classification and presented semantic segmentation networks configured with spiking neurons. Venkatesha et al. (2021) designed a federated learning method to train decentralized and privacy-preserving SNNs. In addition, Kim et al. (2021) proposed PrivateSNN, which aims to build low-power SNNs from a pre-trained ANN model without leaking sensitive information contained in a data set. All these studies inspire the HESFOL model toward applications in other fields, such as federated learning and privacy preservation.

## Conclusion

In this work, we first introduced an entropy-based scheme for SNNs to realize robust few-shot learning performance. We developed a novel spike-based framework with the entropy theory, namely, the HESFOL model, to implement the gradient-based few-shot learning scheme in a recurrent SNN architecture. Several types of tasks are employed to test the few-shot learning performance, including the accuracy and robustness of learning. Experimental results based on spiking patterns, the Omniglot data set, and the motor control task reveal that the proposed HESFOL model can improve the learning accuracy and robustness of the spike-driven few-shot learning performance. The proposed framework offers a novel insight to improve the spike-based machine learning performance based on the entropy theory, which is meaningful for the fast development of brain-inspired intelligence and neuromorphic computing. It can be applied to the unmanned system, neuro-robotic control, as well as edge computing in the Internet-of-Things (IoT).

## Data Availability Statement

The original contributions presented in the study are included in the article/supplementary material, further inquiries can be directed to the corresponding authors.

## Author Contributions

SY developed and tested algorithms, and wrote this manuscript with contributions from BL-B and BC. BL-B and BC conceptualized the problem and the technical framework. All authors contributed to the article and approved the submitted version.

## Conflict of Interest

The authors declare that the research was conducted in the absence of any commercial or financial relationships that could be construed as a potential conflict of interest.

## Publisher’s Note

All claims expressed in this article are solely those of the authors and do not necessarily represent those of their affiliated organizations, or those of the publisher, the editors and the reviewers. Any product that may be evaluated in this article, or claim that may be made by its manufacturer, is not guaranteed or endorsed by the publisher.
